# Efficacy of Shoulder Rehabilitation Post-Cardiac Implantable Electronic Device Implantation

**DOI:** 10.3390/jcm13237014

**Published:** 2024-11-21

**Authors:** Sang-Suk Choi, Yoon-Jin Son, Sung-Jung Kim, Myungjae Yoo, Sumin Roh, Mi-Jeong Yoon, Youmi Hwang

**Affiliations:** 1 Department of Cardiology, St. Vincent’s Hospital, The Catholic University of Korea, Seoul 16247, Republic of Korea; drchoi@catholic.ac.kr (S.-S.C.); zkffh4130@naver.com (Y.-J.S.); kimsj800@naver.com (S.-J.K.); sweetmilkiss@naver.com (M.Y.); soomin2229@gmail.com (S.R.); 2 Catholic Research Institute for Intractable Cardiovascular Disease (CRID), College of Medicine, The Catholic University of Korea, Seoul 06649, Republic of Korea; 3 Department of Rehabilitation Medicine, St. Vincent’s Hospital, The Catholic University of Korea, Seoul 16247, Republic of Korea; allogen@naver.com

**Keywords:** cardiac implantable electronic devices, pain management, patient satisfaction, postoperative care, shoulder function, shoulder rehabilitation

## Abstract

**Background/Objectives**: Cardiac implantable electronic devices (CIEDs) can prevent ventricular arrhythmia-related sudden cardiac death but cause paradoxical discomfort that impairs daily living and quality of life. No management guidelines exist for reducing pain, improving motion around the CIED implantation site, or preventing shoulder contractures. We evaluated the impact of continuous successive shoulder rehabilitation programs for CIED patients on reducing shoulder pain, improving shoulder range of motion, and patient satisfaction in daily living. **Methods**: In this prospective observational study, patients who had received CIED implantation underwent shoulder rehabilitation therapy with education during hospitalization from the day post-CIED implantation. After in-hospital rehabilitation, patients chose to undergo successive shoulder rehabilitation from their home and after 4 weeks in the outpatient clinic (rehabilitation group, n = 68) or not (non-rehabilitation group, n = 33). Pain and shoulder function was assessed using the Visual Analog Scale (VAS) and Disability of the Arm, Shoulder, and Hand (DASH) scores, respectively, the day following CIED implantation and at 1–3 months postoperatively. Patient satisfaction was evaluated using the 36-item Short Form Survey (SF-36). Outcomes were compared between groups. **Results**: VAS pain scores significantly decreased, while DASH scores substantially improved in the rehabilitation group compared to the non-rehabilitation group. Although the SF-36 subdomains were similar between groups, the rehabilitation group showed a better health-related quality of life trend. No complications were observed following post-CIED rehabilitation. **Conclusions**: Shoulder rehabilitation therapy with successive education after CIED implantation significantly improved pain and shoulder function. Definitive guidelines and long-term outcomes should be investigated further.

## 1. Introduction

Cardiac implantable electronic devices (CIEDs) are widely used to manage various arrhythmias, reduce the incidence of sudden cardiac death related to ventricular arrhythmias, as well as reduce the mortality or hospitalization rates in heart failure patients, thereby enhancing quality of life [1,2,3].

While the implantation procedure is relatively quick, living with a CIED can be a lifelong challenge, often accompanied by a range of potential complications.

One complication of CIED insertion is acute to chronic upper-extremity dysfunction, with shoulder pain and movement limitations being the most common manifestations [4]. Many patients experience ongoing shoulder dysfunction after CIED implantation with post-implantation site pain and discomfort due to joint contracture in the shoulder ipsilateral to the implantation site [5]. Shoulder dysfunction after CIED implantation is common and is associated with factors including the pectoral approach to implantation, duration of post-procedure immobilization, hematoma development, and size of the implantable device [4,5,6]. Shoulder dysfunction can lead to long-term disability, impaired quality of life, and adverse psychological effects [7].

No standardized post-CIED implantation management guidelines have been established for preventing shoulder dysfunction, and research on this aspect remains insufficient [8,9,10]. While immobilizing the CIED insertion site could prevent lead displacement, prolonged immobilization time after CIED implantation can lead to stiff shoulder joints, resulting in a limited range of motion and chronic dysfunction. Therefore, maintaining a proper range of motion (ROM) is crucial for improving patients’ quality of life [5,11,12,13,14,15].

Shoulder dysfunction following CIED implantation is a significant clinical concern due to its potential to impair patients’ quality of life and limit daily activities, and shoulder rehabilitation is regarded as effective in preventing shoulder dysfunction [16,17]. However, its efficacy and safety after CIED implantation remain unclear. While previous randomized controlled trials have demonstrated the benefits of rehabilitation programs for preventing musculoskeletal complications, this study suffered from small sample sizes, and the safety of the interventions has not been thoroughly evaluated [14]. Therefore, our study addresses these gaps by systematically evaluating the efficacy and safety of a structured rehabilitation program, aiming to improve functional outcomes, including pain reduction, prevention of joint contractures, patient satisfaction, and informing future clinical guidelines.

## 2. Materials and Methods

### 2.1. Study Design and Protocol

This single-center, prospective observational study investigated the effects of a post-CIED rehabilitation program applied to adult patients over 20 years of age who had newly implanted CIEDs (pacemaker, implantable cardioverter-defibrillator, or cardiac resynchronization therapy) at our institution. This study was approved by The Catholic University of Korea St. Vincent’s Hospital’s Institutional Review Board (Suwon, Republic of Korea; Institutional Review Board No. VC20OISO0250). Inclusion criteria were age > 20 years, CIED implantation at our institution, full comprehension of the informed consent information regarding the rehabilitation program and the study on the day of CIED implantation, and consent to participate. 

In this study, all patients received rehabilitation therapy and education on the day after CIED implantation. After hospital discharge, patients decided whether to continue the rehabilitation program in an outpatient clinic based on their preferences regarding travel distance, transportation, medical costs, and the necessity of guardians. Based on the participants’ choices, the study population was then divided into two groups (rehabilitation [RH] and non-rehabilitation [non-RH] groups). Patients who wished to withdraw from the study or failed to complete the survey within 1–3 months after enrollment were excluded from the study analysis.

**Figure 1:**
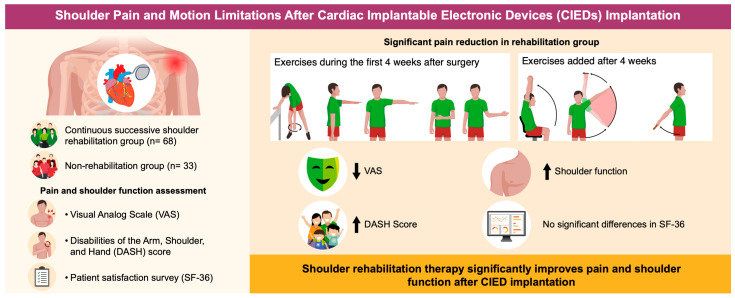
Graphical abstract of rehabilitation program protocol.

The study protocol is summarized in Figure 1. In addition to the baseline assessment, a physical examination of the shoulder joint, a pain survey using a Visual Analog Scale (VAS) score, and the quick Disabilities of the Arm, Shoulder, and Hand (quick DASH) scores were determined for all patients after CIED implantation, during the hospitalization period. Additionally, at 1–3 months post-CIED implantation, a follow-up pain survey (VAS score), patient satisfaction survey using the 36-item Short Form questionnaire (SF-36), and shoulder ROM (range of motion) evaluation using the quick DASH with a physical examination were conducted during outpatient visits. The VAS, quick DASH, and SF-36 scores were assessed using standardized protocols. Surveys were administered face-to-face during hospital visits by trained staff to ensure consistency in responses.

### 2.2. Rehabilitation Protocol

All patients were given a shoulder exercise program starting on the first day after CIED implantation, which was continued for up to 4 weeks at home. The exercises included pendulum and active ROM exercises within a limited range (Figure 1). For the pendulum exercises, the patients were instructed to perform three sets daily with the arm on the side of the pacemaker. Each set involved 10 pendulum movements in the clockwise direction and 10 in the counterclockwise direction. For active shoulder ROM exercises, patients were advised not to raise the shoulder more than 90° during forward flexion and abduction and to perform internal and external rotations with the shoulder adducted without extending the arm backward. Additionally, patients were instructed not to cross their arms behind their backs. They were initially guided to start with five repetitions, three times per day, gradually increasing to a maximum of 10 repetitions per session. The rehabilitation program was conducted under the supervision of trained physical therapists in a dedicated rehabilitation area within the hospital. Patients received detailed guidance on each exercise, and adherence was monitored through exercise logs and follow-up consultations. 

Four weeks after CIED implantation, in the outpatient clinic, the patients were instructed to perform active shoulder ROM exercises in all directions without movement restrictions (Figure 1). Each shoulder ROM exercise was performed once daily and consisted of three sets of 10 repetitions. Compliance with the home-based exercise program was assessed through verbal confirmation during outpatient follow-ups and review of patient-reported adherence logs. Patients who had no difficulty with the exercises after 1 week were instructed to begin performing these while holding a water bottle in their hand. The weight of the bottle started at 0.5 kg and gradually increased based on the individual’s capacity but did not exceed 2 kg. Patients with limited shoulder ROM or pain affecting their daily activities were offered joint mobilization by a physical therapist. Patients with severe pain were treated with analgesics or intra-articular steroid injections.

### 2.3. Outcomes

The primary endpoint consisted of the assessment of shoulder joint function and pain indicators using the VAS and quick DASH scores, measured after device implantation and one session of rehabilitation during hospitalization as well as in the outpatient clinic at 1–3 months after implantation. 

The secondary outcome was patient satisfaction, evaluated through a health status survey using the Korean version of the SF-36. The SF-36 is among the most widely used health-related quality-of-life scales [18]. It is a multifactorial scale including 36 questions and is divided into eight domains for scoring: Physical Functioning (PF), Global Health (GH), Role Physical (RP), Body Pain (BP), Social Functioning (SF), Vitality (VT), Role Emotional (RE), and Mental Health (MH) [19]. Scores ranging from 0 to 100 were assessed for each domain, with higher scores indicating better health. These domain scores ultimately contribute to the evaluation of two composite measures: the Mental Health Score (MHS) and the Physical Health Score (PHS).

### 2.4. Statistical Analysis

For sample size, no similar pilot study has been conducted previously. Therefore, no reference data were available to determine the appropriate sample size. Given that more than 100 CIED procedures were performed annually at our institution between 2019 and 2020, we considered a sample of 100 patients sufficient.

Continuous variables are presented as mean and standard deviation and were compared between the RH and non-RH groups by using *t*-tests or Wilcoxon rank-sum tests, depending on the data distribution. Categorical variables were reported as frequencies and percentages and were compared using the chi-square or Fisher’s exact tests. The primary efficacy outcome, the patient’s assessment of shoulder joint function and pain, was analyzed by comparing pre- and post-rehabilitation results within the same patient group using paired *t*-tests (parametric) or Wilcoxon signed-rank tests (non-parametric). All statistical analyses were performed using R version 3.5.3 (https://www.r-project.org, accessed on 17 August 2024). Statistical significance was defined as a two-sided *p*-value < 0.05.

## 3. Results

### 3.1. Baseline Characteristics

The baseline characteristics of the study participants are summarized in Table 1. The RH and non-RH groups did not differ significantly in terms of sex distribution, age, height, weight, or body mass index (all *p* > 0.05). Similarly, no significant differences were observed in underlying medical conditions, including hypertension, congestive heart failure, sudden cardiac death, stroke, ventricular tachycardia, coronary artery disease, atrial fibrillation, or the use of antithrombotic medication (all *p* > 0.05).

### 3.2. Clinical Outcomes

#### 3.2.1. Primary Outcome

**Figure 2:**
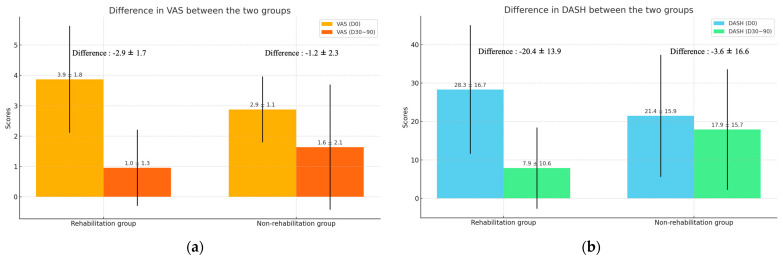
The difference in primary outcome. (**a**) Difference in VAS between the two groups. (**b**) Difference in DASH between the two groups.

The results demonstrated that patients in the RH group experienced significantly greater improvement in both pain relief and shoulder function compared to those in the non-RH group (Table 2). The initial pain scores (VAS(pre)) were higher in the RH than in the non-RH group (*p* = 0.001), but at the 30–90-day follow-up (VAS(post)), the RH group showed a more substantial reduction in pain than that in the non-RH group (*p* = 0.087), with the difference in pain reduction (ΔVAS) being more significant in the RH group (*p* = 0.001) (Figure 2a). Similarly, the initial DASH scores (DASH(pre)) were higher in the RH than in the non-RH group (*p* = 0.053), but the RH group exhibited significantly better shoulder function at follow-up (DASH(post), *p* = 0.002), with a more significant improvement in DASH scores (ΔDASH, *p* < 0.001) (Figure 2b).

#### 3.2.2. Secondary Outcome

The SF-36 results are compared between the RH and non-RH groups in Table 3. In the Physical Functioning (PF) domain, the non-RH group showed slightly higher scores compared to the RH group, but this difference was not statistically significant (68.6 ± 25.2 vs. 63.9 ± 27.4, *p* = 0.468). For the Role Physical (RP) domain, a higher proportion of RH participants achieved a score of 100 (26.4% vs. 12.0% in the non-RH group), suggesting better physical role functioning in the RH group, although the overall difference was not statistically significant (*p* = 0.283). In the Bodily Pain (BP) domain, scores were similar between the two groups (58.6 ± 26.1 in the RH group vs. 54.9 ± 19.1 in the non-RH group, *p* = 0.526). General Health (GH) scores were also comparable (50.5 ± 20.2 in the RH group vs. 48.6 ± 18.9 in the non-RH group, *p* = 0.698). Vitality (VT) scores showed no significant differences between the groups (46.3 ± 20.5 in the RH group vs. 45.8 ± 12.3 in the non-RH group, *p* = 0.890). The Social Functioning (SF) domain showed slightly higher scores in the RH group compared to the non-RH group (64.4 ± 24.1 vs. 59.0 ± 24.3), though the difference did not reach statistical significance (*p* = 0.360). In the RE domain, while 37.7% of the RH group scored 100 compared with 16.0% of the non-RH group, the difference was not statistically significant (*p* = 0.193). MH scores were also similar between the RH and non-RH groups, with no significant difference (59.5 ± 17.1 in the RH group vs. 60.3 ± 12.4 in the non-RH group, *p* = 0.841).

Overall, the Physical Health Score (PHS) and Mental Health Score (MHS) were higher in the RH group compared to the non-RH group, but these differences were not statistically significant (PHS: 42.2 ± 10.6 vs. 41.4 ± 8.8, *p* = 0.765; MHS: 31.4 ± 15.4 vs. 28.2 ± 13.7, *p* = 0.370). 

#### 3.2.3. Safety

Throughout the follow-up period, no lead dislodgement, hematoma, or wound disruption during or after rehabilitation, or reoperations were reported in either the rehabilitation or non-rehabilitation groups.

## 4. Discussion

A short-term immobilization of the shoulder is recommended, at the physician’s discretion, to prevent complications, such as hematoma and lead displacement, in some patients after CIED implantation. However, even short-term post-CIED immobilization or implantation procedures can cause pain or long-term shoulder joint dysfunction, highlighting the need for effective management strategies [4,7,20,21]. We found that a shoulder rehabilitation program for CIED patients significantly enhanced postoperative outcomes, particularly by reducing pain and improving shoulder function recovery. Although differences between the two groups were not statistically significant, patients in the RH group showed better SF-36 PHS and MHS scores. In terms of safety, no cases of lead dislodgement, hematoma, or wound disruption were observed during the shoulder rehabilitation program after CIED. Furthermore, during the study period, some patients who did not undergo rehabilitation therapy after discharge reported shoulder pain and joint contracture a few months later and were diagnosed with adhesive capsulitis, leading to treatments such as intra-articular injections. In contrast, none of the patients in the rehabilitation group reported these issues.

These findings support the results of previous randomized controlled trials [14] and emphasize the importance of early rehabilitation in preventing musculoskeletal complications after CIED implantation [6,10]. Our study further substantiates these findings by demonstrating that patients who participated in the rehabilitation program experienced a greater reduction in pain, as evidenced by the significant decrease in their VAS scores, and showed enhanced shoulder function, as reflected by their improved DASH scores. The reduction in the VAS scores in the rehabilitation group highlights the efficacy of shoulder rehabilitation in managing pain post-CIED implantation. This is particularly relevant given the clinical challenge of balancing the need for immobilization to prevent lead displacement with the risk of shoulder joint contractures due to prolonged immobilization. Our study provided compelling evidence that structured rehabilitation programs can mitigate these risks without compromising device integrity. Furthermore, the significant improvement in the DASH scores in the rehabilitation group highlighted the functional benefits of a rehabilitation program. Such improvement is crucial, as shoulder dysfunction can severely impact the quality of life and potentially lead to long-term disability if not properly managed. The functional recovery observed in our study supports the inclusion of shoulder rehabilitation programs as standard postoperative care for patients with CIEDs. 

Despite its clear value, our study had some limitations. The study was conducted at a single center with a relatively small sample size and used a non-randomized design, which may limit the generalizability of the results. Additionally, the observational design of the study may have introduced a selection bias, as patients who opted for rehabilitation may differ in unmeasured ways from those who did not. In addition, the monitoring of patient adherence to the home-based rehabilitation program is limited, with verbal confirmation and self-reported logs during outpatient follow-ups. Factors such as patient motivation, understanding of the program, and logistical challenges may have contributed to differences in adherence. Future studies should consider incorporating objective measures to more accurately assess adherence and its impact on clinical outcomes.

Another limitation of our study is the possibility of regression to the mean influencing the observed primary outcomes for VAS scores. At baseline, the VAS scores were significantly higher in the rehabilitation group compared to the non-rehabilitation group. This discrepancy could partially account for the greater observed improvement in pain scores in the rehabilitation group, as patients with initially higher scores are more likely to show larger reductions over time. 

Unlike the primary outcomes, the secondary outcome of patient satisfaction using the SF-36 did not show a statistically significant difference, although the RH group displayed better results. The following factors were considered to explain this lack of significance: First, the sample size of the patients included in this study was relatively small, and the patients were older, which may have influenced their responses. As we could not compare the post-procedure SF-36 scores with baseline measurements, we cannot definitively conclude that no improvement in patient satisfaction was achieved based solely on these results.

Subjective assessments, such as the primary and secondary outcomes used in our study, have limitations in quantitative comparisons. 

However, our study provided crucial evidence that supports the need to enhance patients’ abilities to perform daily activities and to improve their satisfaction with long-term care. Based on this finding, we propose that this approach should be considered as a standardized method for post-procedural management of patients who undergo CIED implantation. Future studies should focus on larger multicenter trials to validate our findings and to explore the long-term benefits of rehabilitation on shoulder function and overall patient well-being.

## 5. Conclusions

In conclusion, this study provided clinical evidence supporting the integration of shoulder rehabilitation into the postoperative management of patients who have received CIEDs. The significant improvements in pain and shoulder function observed in the RH group suggest that such interventions should be considered part of standard care protocols. Further research on objective measurements and long-term functional outcomes of CIED rehabilitation programs is warranted.

## Figures and Tables

**Table 1:** Baseline Characteristics of Patients.

Group	Rehabilitation	Non-Rehabilitation	*p*
	(N = 68)	(N = 33)	
Sex			0.104
Male	30 (44.1%)	21 (63.6%)	
Age	69.1 ± 14.4	70.2 ± 10.8	0.684
Height	161.3 ± 10.0	162.8 ± 8.2	0.472
Weight	65.1 ± 13.7	67.3 ± 10.7	0.421
Body mass index	24.8 ± 4.0	25.3 ± 3.3	0.547
CIED			0.435
PM	47 (69.1%)	26 (78.8%)	
ICD	21 (30.9%)	7 (21.2%)	
Atrial hypertension	44 (64.7%)	26 (78.8%)	0.227
Chronic heart failure	14 (20.6%)	4 (12.1%)	0.444
Sudden cardiac death	4 (5.9%)	1 (3.0%)	0.896
Stroke	6 (8.8%)	3 (9.1%)	1.000
Coronary artery disease	10 (14.7%)	8 (24.2%)	0.369
Atrial fibrillation	15 (22.1%)	14 (42.4%)	0.059
Antithrombotic/antiplatelet medication	25 (36.8%)	18 (54.5%)	0.139

CIED, cardiac implantable electronic device; ICD, implantable cardioverter defibrillator; PM, pacemaker.

**Table 2:** Differences in VAS and DASH between the two groups.

Group	Rehabilitation	Non-Rehabilitation	*p*
	(N = 68)	(N = 33)	
VAS(pre)	3.9 ± 1.8	2.9 ± 1.1	0.001
VAS(post)	1.0 ± 1.3	1.6 ± 2.1	0.087
ΔVAS	−2.9 ± 1.7	−1.2 ± 2.3	0.001
DASH(pre)	28.3 ± 16.7	21.4 ± 15.9	0.053
DASH(post)	7.9 ± 10.6	17.9 ± 15.7	0.002
ΔDASH	−20.4 ± 13.9	−3.6 ± 16.6	<0.001

DASH, disabilities of the arm, shoulder, and hand; VAS, visual analog scale.

**Table 3:** Differences in the SF-36 survey between the two groups.

SF-36	Rehabilitation	Non-Rehabilitation	*p*
	(N = 53)	(N = 25)	
PF	63.9 ± 27.4	68.6 ± 25.2	0.468
RP			0.283
0	21 (39.6%)	10 (40.0%)	
25	3 (5.7%)	5 (20.0%)	
50	6 (11.3%)	3 (12.0%)	
75	9 (17.0%)	4 (16.0%)	
100	14 (26.4%)	3 (12.0%)	
BP	58.6 ± 26.1	54.9 ± 19.1	0.526
GH	50.5 ± 20.2	48.6 ± 18.9	0.698
VT	46.3 ± 20.5	45.8 ± 12.3	0.890
SF	64.4 ± 24.1	59.0 ± 24.3	0.360
RE			0.171
0	18 (34.0%)	11 (44.0%)	
33	6 (11.3%)	2 (8.0%)	
67	9 (17.0%)	8 (32.0%)	
100	20 (37.7%)	4 (16.0%)	
MH	59.5 ± 17.1	60.3 ± 12.4	0.841
PHS	42.2 ± 10.6	41.4 ± 8.8	0.765
MHS	31.4 ± 15.4	28.2 ± 13.7	0.370

BP, bodily pain; GH, general health; MH, mental health; MHS, mental health score; PF, physical functioning; PHS, physical health score; RE, role emotional; RP, role physical; SF-36, Short Form 36 Health Survey; VT, vitality; SF, social functioning.

## Data Availability

The data included in this manuscript cannot be shared publicly due to the need to protect the privacy of the included subjects. Data may be shared upon reasonable request to the corresponding author.
